# Correlation of Diffusion and Metabolic Alterations in Different Clinical Forms of Multiple Sclerosis

**DOI:** 10.1371/journal.pone.0032525

**Published:** 2012-03-30

**Authors:** Salem Hannoun, Matthieu Bagory, Francoise Durand-Dubief, Danielle Ibarrola, Jean-Christophe Comte, Christian Confavreux, Francois Cotton, Dominique Sappey-Marinier

**Affiliations:** 1 CREATIS, UMR 5220 CNRS & U1044 INSERM, Université Claude Bernard-Lyon 1, Lyon, France; 2 Service de Neurologie A, Hôpital Neurologique Pierre Wertheimer, Bron, France; 3 Département IRM, CERMEP-Imagerie du Vivant, Bron, France; City of Hope National Medical Center and Beckman Research Institute, United States of America

## Abstract

Diffusion tensor imaging (DTI) and MR spectroscopic imaging (MRSI) provide greater sensitivity than conventional MRI to detect diffuse alterations in normal appearing white matter (NAWM) of Multiple Sclerosis (MS) patients with different clinical forms. Therefore, the goal of this study is to combine DTI and MRSI measurements to analyze the relation between diffusion and metabolic markers, T2-weighted lesion load (T2-LL) and the patients clinical status. The sensitivity and specificity of both methods were then compared in terms of MS clinical forms differentiation. MR examination was performed on 71 MS patients (27 relapsing remitting (RR), 26 secondary progressive (SP) and 18 primary progressive (PP)) and 24 control subjects. DTI and MRSI measurements were obtained from two identical regions of interest selected in left and right centrum semioval (CSO) WM. DTI metrics and metabolic contents were significantly altered in MS patients with the exception of N-acetyl-aspartate (NAA) and NAA/Choline (Cho) ratio in RR patients. Significant correlations were observed between diffusion and metabolic measures to various degrees in every MS patients group. Most DTI metrics were significantly correlated with the T2-LL while only NAA/Cr ratio was correlated in RR patients. A comparison analysis of MR methods efficiency demonstrated a better sensitivity/specificity of DTI over MRSI. Nevertheless, NAA/Cr ratio could distinguish all MS and SP patients groups from controls, while NAA/Cho ratio differentiated PP patients from controls. This study demonstrated that diffusivity changes related to microstructural alterations were correlated with metabolic changes and provided a better sensitivity to detect early changes, particularly in RR patients who are more subject to inflammatory processes. In contrast, the better specificity of metabolic ratios to detect axonal damage and demyelination may provide a better index for identification of PP patients.

## Introduction

Multiple sclerosis (MS) is an inflammatory demyelinating disease of the central nervous system. Characterized by alternating episodes of neurologic disability and recovery [1], this initial relapsing remitting (RR) phase of the disease is frequently followed by a secondary progressive course (SP) expressed by a steady neurological deterioration without remission. Some other forms of MS appear to follow a progressive course from the onset (primary progressive (PP)) without obvious relapses and remissions of neurological deficits [2]. MS pathogenesis implies complex processes, including various degrees of reactive astrogliosis, oligodendroglial loss, phagocytic activity, axonal pathology and some remyelination [3], which lead to focal inflammatory and demyelinating white matter (WM) lesions [4] and diffuse microscopic alterations in normal appearing white matter (NAWM) [5].

Conventional magnetic resonance imaging (MRI) allows a better diagnosis and therapeutic follow-up of MS patients. Typical MRI measures of the disease burden in MS have demonstrated modest correlations with the patients disability. Also known as the “clinico-radiological paradox” [6], this mismatch is probably due to the insensitivity of T2-weighted MRI measures to detect subtle histopathological alterations in NAWM. This argument has contributed to the development of new quantitative MR methods including MR spectroscopic imaging (MRSI) [7] and diffusion tensor imaging (DTI) [8] that present better sensitivity and/or specificity to characterize diffuse alterations in MS.

Based on the measurement of water molecules Brownian motion, DTI has proved to be very sensitive to detect microstructural changes in the WM of MS patients [9]. In lesions, previous studies have shown increased mean diffusivity (MD) and decreased fractional anisotropy (FA) when compared with normal WM of healthy controls. The highest MD values appear to be found in non-enhanced T1-hypointense lesions while the lowest FA values were found in contrast enhanced MS lesions [10]–[11]. These microstructural alterations are probably related to myelin breakdown and/or axonal damage [9] that could be better characterized by the measurement of the diffusion tensor axial (λa) and radial (λr) diffusivities [12]–[13].

Proton MRSI provides the spatial distribution of several metabolites including N-acetylaspartate (NAA) [14], which is associated with axonal/neuronal damage or dysfunction [15]). While the creatine (Cr) signal provides an index of cell proliferation by monitoring cellular energy metabolism, the choline (Cho) signal constitutes a specific marker of membrane metabolism, making it sensitive to demyelination and remyelination processes. In active inflammatory MS lesions and NAWM, metabolic alterations included increases in Cr and Cho content and variable decreases of NAA concentration. In contrast, NAA reduction was more severe in chronic non-enhancing lesions confirming its ability to detect axonal damage [16]. Finally, these metabolic measures proved to be better correlated with the patient clinical status than conventional MRI [17].

Therefore, we first proposed in this study to analyze the relation between microstructural changes and metabolic alterations, measured by DTI and MRSI respectively, in the centrum semioval (CSO) region of MS patients with different clinical forms. Second, correlations between these MRI markers, the T2 lesion load (T2-LL), and the patient clinical status were analyzed to better understand the underlying pathological processes and their clinical expression. Third, DTI and MRSI methods were compared in terms of sensitivity and specificity, for the detection of pathological changes and the differentiation of MS clinical forms.

## Materials and Methods

### Subjects

Seventy-one patients (27 RR, 26 SP and 18 PP) with clinically definite MS according to the McDonald's criteria [18] were included in this study (Table 1). All patients underwent a full neurological examination performed by a neurologist, including the measurement of their expended disability status scale (EDSS). This work is part of an observational longitudinal study in which medical therapy has been unchanged. Thus, only 22% of PP patients versus 63% and 58% of RR and SP patients, respectively, were under immune-modulating therapy. Twenty-four volunteers with no history of neurological disorders served as control subjects. Local ethical committee approval and written informed consents were obtained from all patients and control subjects prior to study initiation.

**Table 1:** Demographics and group characteristics.

	Controls	RR	SP	PP	All-MS
**Number of subjects**	24	27	26	18	71
**Age [years]**	36.0 (9.5)	35.1 (7.3)	41.7 (5.1)	41.2 (6.5)	39.1 (7.0)
**Disease duration [years]**	-	6.9 (4.2)	14.3 (6.1)	6.7 (3.1)	9.5 (6.0)
**Median EDSS (range)**	-	2 (0.0–6.0)	4.8 (2.5–7.0)	4.0 (2.5–7.0)	4.0 (0.0–7.0)
**ROI T2-LL (%)**	-	19.7 (11.3)	28.9 (15.2)	39.0 (20.6)	28.0 (17.1)
**Brain T2-LL (%)**	-	0.14(0.09)	0.29(0.24)	0.61(0.33)	0.60(0.41)

Values are expressed as mean (Standard deviation).

RR: relapsing remitting; SP: secondary progressive; PP: primary progressive; EDSS: expanded disability status scale; ROI T2-LL: T2-lesion load corresponding to the lesion volume measured within the region of interest (ROI); Brain T2-LL: T2-lesion load corresponding to the lesion volume measured in the whole brain.

### MRI Protocol

All patients and control subjects underwent MR examination using a 1.5 Tesla MR system (Sonata Siemens, Erlangen, Germany) and an 8 elements phased-array head-coil. Conventional MRI protocol comprised a 3 dimensional T1-weighted (magnetization prepared rapid gradient echo-MPRAGE) sequence with and without gadolinium injection (repetition time/echo time/time for inversion [TR/TE/TI] = 1970/3.93/1100 ms; flip angle = 15°; matrix size = 256×256; field of view [FOV] = 256×256 mm; slice thickness = 1 mm; voxel size = 1×1×1 mm; acquisition time = 4.62 min), a Turbo Spin-Echo (TSE) with proton density (PD) and T2-weighted contrasts (TR/TE1/TE2 = 3020/12/85 ms; flip angle = 180°; matrix size = 192×256; FOV = 240×240 mm; slice thickness = 3 mm; voxel size = 0.9×0.9×3 mm; acquisition time = 4.55 min) and a fluid-attenuated inversion recovery (FLAIR) sequence (TR/TE/TI = 8000/105/2200 ms; flip angle = 150°; matrix size = 192×256; FOV = 240×240 mm; slice thickness = 3 mm; voxel size = 0.9×0.9×3 mm; acquisition time = 4.57 min).

A spin-echo echo-planar DTI sequence was performed with the following imaging parameters (TR = 6900 ms; TE = 86 ms; matrix size = 96×96; FOV = 240×240 mm; slice thickness = 2.5 mm; voxel size = 2.5×2.5×2.5 mm; acquisition time = 6.67 min) to acquire 51 axial slices oriented along the anterior commissure-posterior commissure (AC-PC). Diffusion gradients were applied along 24 non-collinear directions with a b factor of 1000 s/mm^2^. The b0 image was acquired four times to increase signal to noise ratio.

MRSI acquisition consisted in one slice placed above the corpus callosum along the AC-PC axis, encompassing the centrum semioval region. A PRESS sequence (TR = 1690 ms; TE = 135 ms) was used to select a VOI of 10×10×1.5 cm during the acquisition of 24×24 (interpolated to 32×32) phase-encodings over a FOV of 240×240 mm.

### Data Analysis and Post-Processing

All image post-processing was performed on a Windows PC workstation. For correlation purpose between metabolic and diffusion measures, two identical regions of interest (ROI) were selected in left and right CSO areas from MRSI and DTI data.

First, two ROIs of 6×2 voxels were selected from the MRSI. MR signals obtained from the 24 voxels were automatically corrected for phase and frequency shift using residual water signal and summed to produce a single MR signal for each subject. Water signal was suppressed by a Singular Value Decomposition of a Hankel matrix using Lanczos algorithm (HLSVD) using 25 components on a frequency window from -2.5 to 0.46 ppm. Background signal from lipids, macromolecules and lineshape distortions were handled using a subtract method of first temporal points optimized to 5 ms. Signal amplitude of metabolites was finally estimated by the QUEST algorithm [19] with prior information of NAA, Cho and Cr simulated by NMRScope [20]. Metabolic ratios were calculated, and concentrations obtained using a calibration on a reference phantom of known concentrations [21].

Second, the ROIs previously selected from MRSI were positioned on diffusion maps based on their absolute coordinates. From each ROI corresponding to 15×5×5 voxels of DTI images, histograms were calculated to extract the peak position of each diffusion metrics. MedINRIA software (http://www-sop.inria.fr/asclepios/software/MedINRIA/) was used to calculate and visualize FA, MD, λa (λ1) and λr (mean of λ2 and λ3) maps.

The ROI T2-LL value was obtained by measuring the volume of the manually segmented T2-lesions inside the ROI. The brain T2-lesion volume measurements were extracted from co-registered T1-, T2-, PD-weighted and FLAIR images, using an automatic segmentation algorithm in the SEPINRIA software [22]. The lesion masks were validated by an experienced neurologist before calculating the lesion volume. ROI and brain T2-lesion volumes were then normalized by the ROI volume and the subject intracranial volume, respectively.

### Statistical Analysis

Statistical analysis of diffusion and metabolic results was performed using Data Analysis and Statistical Software (STATA, Version 9.2, StataCorp, Texas, USA). Metabolic concentrations and ratios, and diffusion values were compared between patients and controls using a One-Way ANOVA test. Bonferroni correction was used to perform post-hoc multiple comparisons. Spearman correlation test was applied to assess the relationship, first between diffusion metrics and metabolic concentrations and ratios (corrected for ROI T2-LL), and second between DTI and MRS measures along with T2-LL and EDSS values. Correlations were not corrected for multiple comparisons. Values were considered significant at p<0.05.

Comparison of DTI and MRSI efficiency to differentiate MS patients from control subjects was performed using a receiver operating characteristic (ROC) curve analysis. Sensitivity and specificity of the most significant DTI metrics (MD, λr) were compared to those of MRSI (NAA/Cr, NAA/Cho) by analyzing the areas under ROC curves (AUC) in different clinical forms (RR, SP, PP) and in the all-MS patients group *versus* control subjects.

## Results

### Diffusion measures

DTI-derived parameters measured in the CSO regions showed significant alterations in every MS patients group (Table 2). Increases of both axial and radial diffusivity values, with λr changes (21.0%) being twice as much higher as λa (11.2%) in all-MS patients group, resulted in highly significant MD increases and FA reductions. When comparing SP to RR patients, FA values were significantly decreased (p<0.01) whereas MD (p<0.01), λa (p<0.05) and λr (p<0.001) values were increased. In contrast, no significant changes were detected between PP and RR patients.

**Table 2:** Diffusion and metabolic measures in MS patients and control subjects groups.

	Controls	RR	SP	PP	All-MS
**MD**	0.71±0.03	0.79±0.05***	0.86±0.10***	0.83±0.09***	0.83±0.09***
**FA**	0.40±0.02	0.38±0.03*	0.35±0.03***	0.36±0.04***	0.36±0.04***
**λa**	1.03±0.04	1.12±0.05***	1.18±0.10***	1.16±0.10***	1.15±0.09***
**λr**	0.55±0.03	0.63±0.05***	0.70±0.10***	0.67±0.09***	0.67±0.09***
**NAA**	11.52±1.01	11.12±0.76	10.19±1.29***	10.20±1.25***	10.54±1.18***
**Cho**	1.52±0.17	1.61±0.17	1.66±0.35	1.66±0.20	1.64±0.25*
**Cr**	9.26±0.90	9.60±0.80	9.89±0.93*	9.67±0.84	9.72±0.85*
**NAA/Cr**	2.54±0.15	2.37±0.18**	2.10±0.25***	2.15±0.18***	2.22±0.24***
**NAA/Cho**	2.72±0.28	2.49±0.31	2.28±0.50***	2.21±0.30***	2.34±0.40***

Values (Mean ± SD) of mean diffusion (MD), fraction of anisotropy (FA), axial (λa) and radial (λr) diffusivities, N-acetylaspartate (NAA), choline (Cho) and creatine (Cr) (*p<0.05; **p<0.01; ***p<0.001 when compared to controls). RR: relapsing remitting; SP: secondary progressive; PP: primary progressive.

### Metabolic measures

Quantification of metabolite concentrations showed a significant decrease of NAA concentration, and increases of Cr and Cho concentrations, in the all-MS patients group compared to controls (Table 2). While there were no significant changes in RR patients compared to controls, NAA concentrations were significantly reduced in SP and PP patients. Cr concentrations increased significantly only in SP patients. The estimation of these concentrations resulted in significant decreases of NAA/Cr and NAA/Cho ratios in the all-MS patients group as well as in SP and PP patients relative to controls. NAA/Cr ratios were also significantly lower in RR patients compared to controls.

### Relationship between diffusion and metabolic measures and T2-lesion load

Diffusion parameters showed significant correlations with NAA concentrations and NAA/Cr ratios in MS patients, either pooled all together or separated by clinical forms (RR, SP and PP) (Table 3). Interestingly, NAA was only significantly correlated with the axial diffusivity in RR patients. Also, NAA/Cho ratios were significantly correlated with FA values in PP patients (r = 0.69, p<0.01).

**Table 3:** Correlation rates (r) between diffusion and metabolic measures corrected for T2-lesion load in different groups of MS patients.

	NAA/Cr				NAA			
	RR	SP	PP	All-MS	RR	SP	PP	All-MS
**MD**	−0.60***	−0.53**	−0.56*	−0.61***	−0.37	−0.53**	−0.57*	−0.57***
**FA**	0.54**	0.64***	0.46	0.58***	0.29	0.50**	0.67**	0.56***
**λa**	−0.52**	−0.47*	−0.55*	−0.57***	−0.39*	−0.50**	−0.41	−0.51***
**λr**	−0.60***	−0.55**	−0.54*	−0.61***	−0.35	−0.54**	−0.63**	−0.59***

Correlation coefficient (*p<0.05; **p<0.01; ***p<0.001).

MD: Mean diffusion, FA: fraction of anisotropy, λa: axial diffusivity, λr: radial diffusivity, NAA: N-acetylaspartate, Cr: creatine, RR: relapsing remitting; SP: secondary progressive; PP: primary progressive.

**Figure 1:**
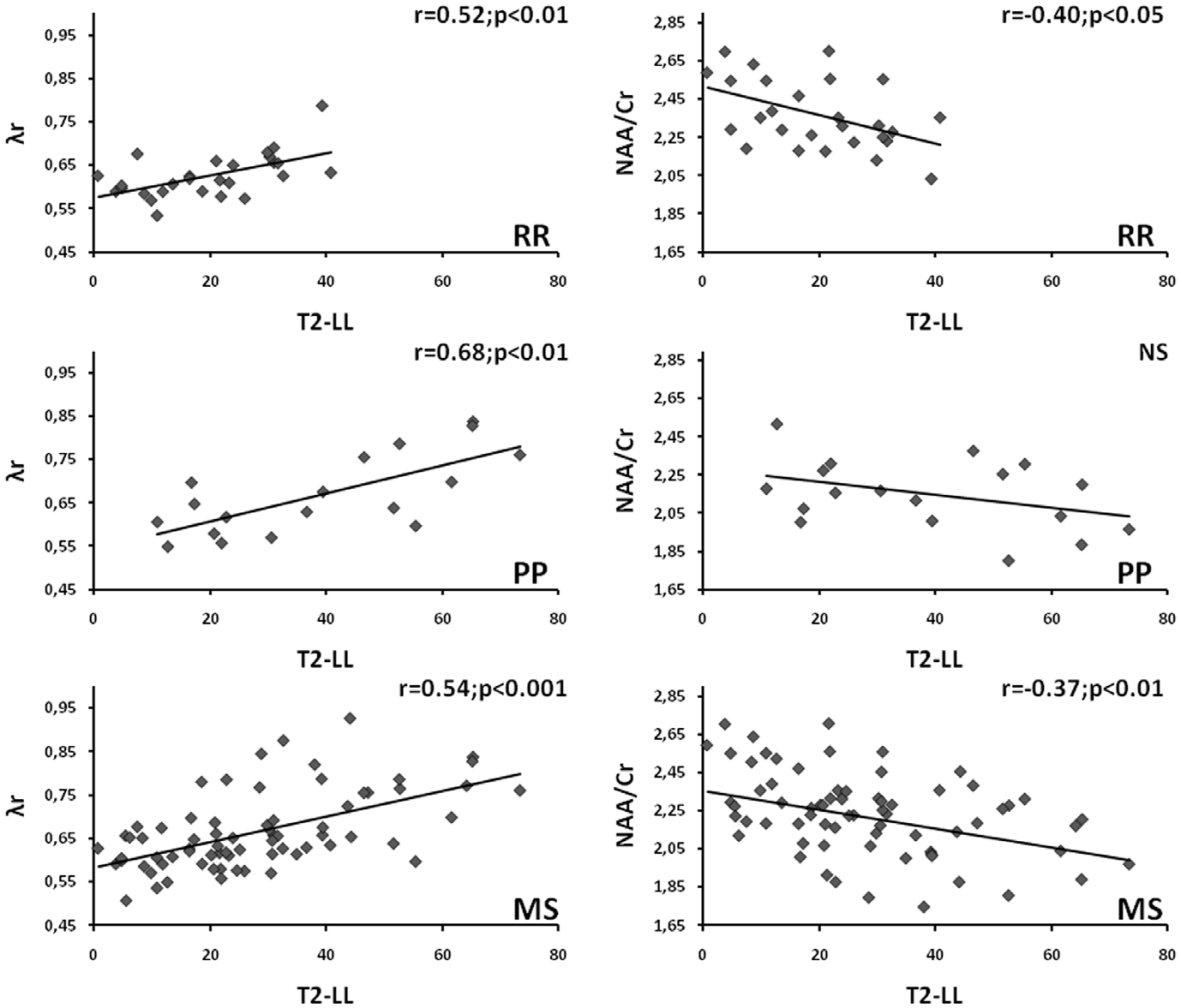
Correlations between radial diffusivity (λr) and NAA/Cr ratio with the ROI T2-LL in RR, PP and all-MS patient groups. (NS = Not significant).

In the all-MS patients group, significant correlations were observed between the ROI T2-LL and FA (r = −0.29; p<0.05), MD, λa, λr, NAA concentration (r = −0.31; p<0.01) and NAA/Cr ratio (Table 4). When comparing clinical forms, correlations between the ROI T2-LL and MD, axial and radial diffusivities were the most significant in PP patients. In contrast, the NAA/Cr ratio and the NAA content were not correlated with the ROI T2-LL in any patients groups, with the exception of the NAA/Cr ratio in RR patients. To illustrate these results, the correlations between radial diffusivity and NAA/Cr ratio with the ROI T2-LL were displayed in Fig. 1 for RR, PP and all-MS patient groups.

**Table 4:** Correlation rates (r) between diffusion and metabolic measures and the ROI T2-LL in different groups of MS patients.

	ROI T2-LL			
	RR	SP	PP	All-MS
**MD**	0.62***	0.42*	0.71***	0.58***
**λa**	0.56**	0.50**	0.72***	0.61***
**λr**	0.52**	0.39*	0.68**	0.54***
**NAA/Cr**	−0.40*	−0.08	−0.33	−0.37**

Correlation significance (*p<0.05; **p<0.01; ***p<0.001).

ROI T2-LL: T2-lesion load corresponding to the lesion volume within the region of interest, MD: mean diffusion, FA: fraction of anisotropy, λa: axial diffusivity, λr: radial diffusivity, NAA: N-acetylaspartate, Cr: creatine, RR: relapsing remitting; SP: secondary progressive; PP: primary progressive.

Regarding the association with the patients disability measured by the EDSS, both diffusion (FA: r = −0.34, p<0.01; MD: r = 0.25, p<0.05; λr: r = 0.28, p<0.05) and metabolic (NAA/Cr: r = −0.36, p<0.01) measures were moderately correlated in the all-MS patient group. EDSS was also correlated with FA values (r = −0.52; p<0.05) and several metabolic measures (NAA/Cho: r = −0.50; p<0.05; Cho/Cr: r = 0.48; p<0.05; Cho: r = 0.55; p<0.05) in PP patients. No significant correlations were found between EDSS and T2-LL (brain or ROI).

### Analysis of ROC Curves

**Figure 2:**
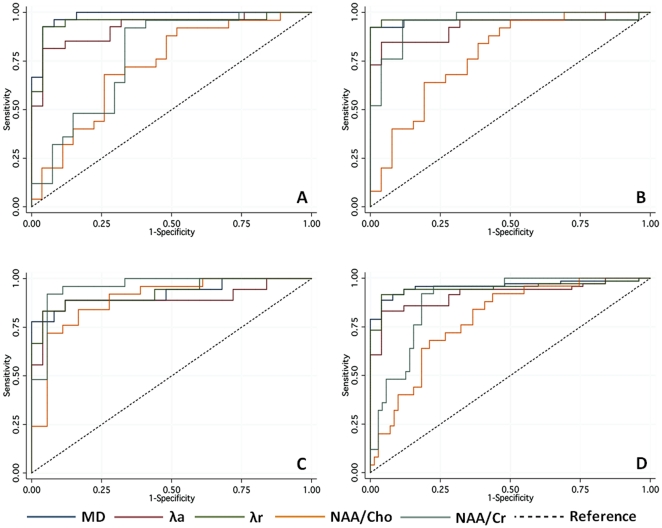
ROC curves of the mean diffusion (MD), axial (λa) and radial (λr) diffusivities, NAA/Cho and NAA/Cr ratios in RR (A), SP (B), PP (C) and all-MS patient groups (D).

ROC analysis was performed to compare the efficiency of diffusion and metabolic markers to differentiate controls from MS patients, taken as one group or separated by clinical forms (Fig. 2). If the overall best sensitivity/specificity was obtained by MD and λr measures, the NAA/Cr ratio was also efficient to distinguish all-MS and SP patient groups from controls. NAA/Cho ratio also helped to better differentiate PP patients from controls (Table 5). However, the metabolic ratios of NAA/Cr and NAA/Cho were significantly less efficient than diffusion metrics to distinguish RR patients from controls.

**Table 5:** Areas under ROC curves analysis of DTI and MRSI derived measures in different groups of MS patients.

	RR	SP	PP	All-MS
**MD vs. NAA/Cr**	0.98>0.78**	0.96≈0.96	0.92≈0.96	0.96≈0.89
**MD vs. NAA/Cho**	0.98>0.72***	0.96>0.78*	0.92≈0.89	0.96>0.79**
**λr vs. NAA/Cr**	0.95>0.78*	0.96≈0.96	0.93≈0.96	0.95≈0.89
**λr vs. NAA/Cho**	0.95>0.72**	0.96>0.78*	0.93≈0.89	0.95>0.79**

Statistical significance (*p<0.05; **p<0.01; ***p<0.001) when comparing areas under ROC curves (AUC) of the following MR metrics: MD: mean diffusivity, λr: radial diffusivity, NAA: N-acetylaspartate, Cr: creatine, RR: relapsing remitting; SP: secondary progressive; PP: primary progressive.

## Discussion

In this study, diffusion and metabolic parameters were measured in the centrum semioval WM region of MS patients with different clinical forms in order to test first, their inter-relations and second, their correlations with the T2-LL and the patient clinical disability. Both MR modalities provided several metrics that were significantly modified by the pathological processes occurring in MS. Further, these diffusion and metabolic changes were significantly correlated highlighting a relation between microstructural and metabolic alterations.

If all diffusion parameters presented a high sensitivity to detect alterations in any clinical form, and even in the less severe RR form, one can notice the greater change of λr compared to λa. As reported in animal models of MS [23], these changes may reflect greater damage in myelin [12] than in axons [24]–[25]. Indeed, the correlations observed between the diffusion metrics and the ROI T2-LL highlighted a link between local injuries due to inflammatory and/or demyelinating lesions [26] and microstructural alterations. Furthermore, these correlations were more significant in RR and PP patients that are more subject to inflammatory processes than SP patients.

Metabolic alterations found in MS patients included increases of Cr and Cho concentrations and reduction of NAA content leading to decreased NAA/Cr and NAA/Cho ratios. In agreement with previous studies [16], these findings showed that NAWM is subject to several tissue restructuring. While the reduction of NAA, a compound present in neurons [27], may reflect axonal damage, Cr increase was reported as a putative marker of cell proliferation that is consistent with oligodendrocytes remyelination and astrocytic gliosis [28]. In contrast to diffusion, metabolic measures showed no significant correlation with the ROI T2-LL, except for the NAA/Cr ratio in RR patients. These findings may suggest that NAA changes are less sensitive to inflammatory processes occurring in T2-lesions but might be more specific to irreversible axonal damage. This hypothesis was furthermore highlighted by the correlation found between NAA concentration and axial diffusivity in RR patients. Alternatively, the correlations obtained between NAA/Cr ratios and T2-LL values were only significant in RR patients reflecting the association of subtle axonal abnormalities and cell proliferation, probably due to indirect effects of inflammation [29].

Correlations between diffusion metrics and NAA/Cr ratios were significant in every MS patient group, especially in PP patients. Such correlations between reduced NAA values and increased MD were previously reported in the WM of PP patients [30]. Also, Oh et al. [31] reported moderate correlations between radial diffusivity and NAA/Cr ratio measured in the corpus callosum of MS patients. Thus, these correlations observed between diffusion metrics and NAA/Cr ratios highlighted a putative link between microstructural and metabolic alterations that could both be the resultant of the inflammatory cascade and demyelinating processes, and/or axonal damages. If the degree of correlation varied with the patient clinical status, the most significant result was found in PP patients where both degenerative and inflammatory mechanisms are believed to occur simultaneously [32].

In contrast, correlations measured between diffusion metrics and NAA were only significant in the progressive forms of MS (SP and PP), and particularly in SP patients. These findings probably revealed the better specificity of NAA to reflect chronic lesions and the underlying axonal loss. This interpretation is reinforced by the lack of correlation in RR patients where NAA changes may represent reversible metabolic dysfunctions or axonal integrity rather than irreversible axonal transection [26]. RR patients are probably more subject to reversible inflammatory and remyelinating mechanisms according to their best response to anti-inflammatory treatments [33]–[35]. Indeed, the lack of significant NAA changes observed in our RR group could result from the immune-modulating medication prescribed to most of these patients.

Significant correlations between FA and NAA/Cho ratio were also observed in PP patients suggesting that the NAA/Cho ratio could constitute a specific index of primary pathological mechanisms including demyelination and axonal damage [36]. Indeed, the ROC analysis showed that the NAA/Cho ratio was as efficient as diffusion metrics and NAA/Cr ratio, to differentiate PP patients from control subjects. Nevertheless, MD and λr measures were significantly more sensitive than any metabolic ratios to differentiate RR patients from controls, suggesting that diffusion metrics constitute the most sensitive indicators of early inflammation-related structural damage in NAWM.

In conclusion, the analysis of the relation between microstructural damage (as detected by DTI) and metabolic alterations (as detected by MRSI) demonstrated the better sensitivity of diffusion relative to metabolic markers. This sensitivity to early inflammatory processes occurring in RR patients was evidenced by the correlation observed between diffusion markers and the T2-LL. In contrast, metabolic ratios such as NAA/Cr or NAA/Cho may constitute efficient markers, due to their better specificity, to differentiate progressive forms where neurodegeneration is more pronounced. Therefore, the complementary sensitivity of DTI with the specificity of MRSI to detect early brain microstructural changes along with diffuse metabolic alterations may provide a better understanding of the various pathological processes interactions.
