# Correction: Correlation of Diffusion and Metabolic Alterations in Different Clinical Forms of Multiple Sclerosis

**DOI:** 10.1371/annotation/beed29e2-e61a-4d2f-8baa-ac53cf2fee7b

**Published:** 2012-08-06

**Authors:** Salem Hannoun, Matthieu Bagory, Francoise Durand-Dubief, Danielle Ibarrola, Jean-Christophe Comte, Christian Confavreux, Francois Cotton, Dominique Sappey-Marinier

The following information was missing from the Funding section: This research was also sponsored by the Région Rhône-Alpes and the public grant from the French "Agence Nationale de la Recherche" within the context of the "Investments for the Future" program, referenced ANR-10-COHO-002. 

